# Public Health Questions in the United States—Extract from Address

**Published:** 1877-03

**Authors:** J. M. Toner


### Public Health Questions in the United States.

Extract from Address. BY J. M. TONER, M. D.

Men live and carry on all their various avocations in an atmosphere only a few feet above the earth. A more important and comprehensive record of the actual meteorological phenomena occurring in the limited belt or sphere of man's activities, might be obtained, if all observations of temperature, moisture, rainfall, electricity, etc., were taken close to the earth. I allude to the subject with a view to encourage an exact uniformity of elevation and exposure of instruments in making observations. We are particularly interested in the condition of the atmosphere we breathe, and should desire to know its exact conditions and extremes of variations. It is a well-ascertained fact, that the rain-gauge registers in the same locality more at the earth's surface than when placed at a considerable height. Changes of temperature, too, are effected by elevation, by radiation from the earth according to the seasons, and during the different hours of day and night. The thermometer ought, in my opinion, to be placed close to the earth, and never more than five feet above it, and be remote from buildings shaded from the direct rays of the sun; but exposed to the free currents of air, though cut off from the radiated heat from the ground.

We are gratified to observe that the importance of hygiene and sanitary inquiries engaged the attention of the last Congress of the United States, which passed an act authorizing the Surgeon-general of the Army, and the chief of the Marine Hospital Service, to investigate the history and cause of the cholera epidemic of 1873, and of the yellow fever epidemic during the same year—the first named to report on cholera, and the latter on yellow fever. The researches of these departments have resulted in the publication of valuable reports, which have added much to our knowledge of the history and characteristics of these diseases. Congress, too, with an awakening sense of its duties and responsibilities, is beginning to consider the subject of the food supply of the people. This indeed is a most important question to every government. An Act of Congress of 1873, authorized the organization of a "United States Commission of Fish and Fisheries;" the labors of this commission are chiefly directed to an investigation of the food fishes of the American coast and rivers.

This action of the government suggests an extension of the principle to other matters intimately connected with the obligation to consider all the conditions that relate to feeding, clothing, housing, and the general well-being of the people.

The meteorological observations being collected so systematically, and from all parts of the country, by the United States Signal Office, present a vast field for the study of the influence of climate, storms, and the like, upon health. The facts collected by this department are yearly coming to be more sought after by the physician. There is no occupation or mode of life that does not deserve to be investigated as to its influence upon health. Man's surroundings, his domicile, his clothing, his food, his habits, in childhood, in mature years, and in old age, all should be observed, with a view to discover the conditions which develop the highest vigor of body and mind, and secure the greatest longevity. We assume that life is a blessing. To preserve it is a duty, not a virtue. No circumstance is, therefore, trivial, that can unfavorably affect health or shorten our existence. Man's social instinct and moral nature in a measure make him "his brother's keeper." It is surely, therefore, a natural, if not a Christian duty, resting upon all persons possessing the knowledge,—to point out the physical evils which flow from bad habits, and from the neglect of hygiene.

The statement is often made, and is generally believed, that residents of rural districts enjoy a higher average degree of health, physical strength, and a greater longevity than those who live in cities. If this opinion be correct, what are the conditions that secure such results? I am inclined to think that regular habits, as well as fresh food and pure air, have much to do in the attainment of this higher degree of vitality which common belief assigns to rural life. A philosopher says: "Habits makes the man." One of the most notable differences in the habits of the two is in the hours chosen for retiring. Country people undoubtedly sleep more, and have fewer disturbances during the night than the residents of cities. Can it be that peaceful lives, with abundance of sleep, are the chief factors in securing vigor, good health, and longevity? It seems probable that such is the case. Examples in confirmation of this view, will, I believe, occur to every one. The want of sufficient sleep at the proper time is, in my opinion, a fruitful cause of disordered functions and impaired health.

It is not likely that any suggestions of mine will lead to reform in this particular, but it is not, therefore, the less a duty to point out what contributes to vicious habits, and indicate the causes that are probably involved in the deterioration of the vital forces.

As conducing to an amendment in this regard, I would suggest that in cities all licensed restaurants and bars should be required by law to close at ten o'clock, and that theatres, and other places of amusements, commence at seven o'clock, so as to close at ten, or eleven at the latest. If it were possible to add one hour each night to the sleep of the residents of cities, I feel persuaded it would do much to elevate morals and preserve health.

A great American statesman is reported to have said that large cities are the plague spots of nations. Whether this be true or not in a political sense, there are many who believe that cities are in danger of becoming emenable to the charge in a sanitary point of view. The overcrowding that always takes place is to be deplored, and deserves the attention of statesmen and legislators. Large aggregations of people are constantly in a condition to be surprised by some contagious disease. Is it not reasonable that health organizations should in some way favor cheap transportation from cities into the country, so as to relieve the crowding of tenement houses. A measure of this kind, with a homestead exemption law, which would secure a cheap home even thirty miles from the city, with a lot for a garden to the head of a family, might do something to encourage a considerable number to live in the country, who now crowd into all sorts of abodes in large cities.

A homestead exemption law would probably give great encouragement to the poor of economic disposition, and who would take advantage of it for the sake of a home where they might hope to raise their children to adult life, to do which is next to impossible in crowded cities. A law of this nature, or any other measure that would multiply small homes, and encourage rural life and garden culture, ought to be welcomed, since it is a recognized principle in political economy that the greater the number of small land-owners, the greater the strength and stability of a nation.

Among the multitude of questions that press for consideration, there is one upon which I wish to say a word, as I understand it to be a usage, in some localities, and which I am sure all will agree in condemning as heartless and inhuman, namely, the practice of not removing a drowned person from the water immediately on discovery. Man's better instincts revolt at so barbarous a custom. I am informed that the opinion prevails, that individuals render themselves liable to prosecution if they meddle with a drowned body, even to attempt resuscitation, until the coroner arrives and assumes control. Such delay and inference as to any law upon the subject ought not to be sanctioned by our silence, as it is contrary both to the dictates of humanity and public policy. In cases of recent drowning, not a moment should be lost in removing the body from the water. It is well known that many lives have been saved even in cases of apparently complete drowning, by prompt, intelligent and persistent efforts at resuscitation. When a body that has not been longer than thirty or forty minutes under water can be recovered, attempts to effect a revival should be immediately commenced, by all such manipulations as will imitate natural breathing, whether in a boat or on shore, and they should be continued for more than half an hour. Any neglect of this duty ought to be esteemed highly reprehensible.

If the health and police regulations of cities having water fronts, do not contain laws in accordance with these principles, they must, I think, be considered defective, and should at once be amended. The sentiment, too, ought to be widely inculcated among all classes that it is man's imperative duty in every case of recent drowning to instantly remove the body from the water and to make every endeavor to induce resuscitation.

A Commission on Forestry for the United States was discussed at the last Congress, but failed to become a law, though the expediency of establishing some means to preserve the natural forests from wanton or negligent destruction, as well as the foundation of a systematic and comprehensive plan for planting trees, was fully brought before the public, in the discussion in the House of Representatives, and particularly in the report upon the subject made by the "Committee on the Public Lands," which recommended the enactment of a law for the appointment of a commission.

It is generally conceded that the rapid exhaustion of the great forests of our country has wrought important changes in our climate, disturbing the regular and equal distribution of rain.

Too much importance cannot be attached to the planting and preservation of trees, not only in the country but along all streets, and in every practicable locality in cities. Their vigorous grcwth along streets will shield from the glare of the sun, and if numerous, preserve by their refrigeration a slightly lower temperature in summer, and lessen the amount of dust on thoroughfares, and thus add to comfort and health during the heated term. The question of the importance of preserving timber for climatic, economic, and hygienic purposes will, it is understood, be presented to this Association by a gentleman who has given great attention to the subject.

Our country during the past year has been free from severe epidemics. Yellow fever appeared in some of our seaport cities. At Key West, and at Pensacola, Fla., where the government maintains both a military and a naval station, this disease appeared; also at Milton, Fla., at West Pascagoula, Miss., and at Howell's Station, twenty-five miles above Pensacola; and at Mobile, Ala., and at New Orleans, La. It was also taken to Brooklyn, N. Y., but by energetic measuıes of quarantine, and other hygienic and sanitary measures, the *scourge of the Gulf seaports* was suppressed wherever it appeared, not, however, until a number of deaths had occurred.

The Marine Hospital Service, and the local government authorities in the South, have been working harmoniously and efficiently together during the past season under an old law of the United States, requiring the co-operation of the revenue and military forces with the State health or quarantine officers for the protection of the public health.

Among the preventable diseasəs, diphtheria has been fatal to a degree that attracts special attention in the mortuary reports of the cities of New York, Brooklyn, Jersey City, Philadelphia, St. Louis and Boston. Small-pox, too, has persisted in claiming an excessive pereentage of deaths in the cities of New York, Brooklyn, St. Lonis, Cincinnati, and other places.

Next to the sewerage and drainage in its importance to health, we may place the lighting and ventilation of dwellings. Few others than physicians and health inspectors, who are called upon to attend the poor in large cities, crowded into tenement-houses, cellars, and garrets, can realize to what extent the breath, perspiration, and other excretions from the human body, detained in clothing or apartments, poison the atmosphere in which these people live. Often people of this class are so crowded together, that they are slowly but surely becoming their own executioners, and with poisons excreted from their own bodies. It is not generally realized that the excreta from the lungs and skin are greater in weight, for the twenty-four hours, than those from the kidneys and bowels.

